# M. Bricheteau on the Pathology of Asthma

**Published:** 1826-07

**Authors:** 


ON THE DISEASE CALLED ASTHMA.

It is well known that some French authors have endeavoured, of late, to blot the term, asthma, out of the nosological chart, attributing the phenomena of that disease to organic affections of the heart, great vessels, or lungs. It is probable that this is the true pathology of asthma in many cases, but certainly not in all, or even in the majority. The following observation is by M. Bricheteau, an able pathologist of the French capital.

*Case.* M. S. a Counsellor, a young man, 28 years of age, of nervous temperament, passed for an asthmatic in his native town, and was treated as such by his physicians. He had habitual dyspnœa, aggravated periodically. In the month of January, 1825, he came to Paris on business, and there he got much worse, in consequence of some altercations and mental perturbations. M. Bricheteau was sent for on the 15th January, and found him labouring under much difficulty of breathing, accompanied by quickness and irregularity of pulse. Yet in the midst of this dyspnœa and laborious exertion of the respiratory muscles, the skin was cool, and his countenance calm. He had cough at intervals, with some sanguineous expectoration. The action of the heart being irregular and tumultuous. The chest was sonorous in almost every point, and when examined by the ear and by the stethoscope, presented the phenomenon of loud wheezing every where except for some space at the lower part of the left side, where the respiratory murmur was scarcely audible. The patient was obliged to keep constantly sitting upright, and changing his position. He had not slept for several nights. He refused to loose blood from the arm, but, with difficulty, consented to the application of twenty leeches to the least sonorous part of the chest. Toast water, with nitric ether, and pediluvia with mustard. Next day the patient was better. He had slept several hours in the night; but, on the 17th, the complaint increased greatly, and the sense of suffocation was imminent, the act of inspiration being performed with great difficulty, and, as it were, convulsively. He asserted that he must be suffocated if not relieved. The pulse was extremely frequent and irregular, as were the pulsations of the heart—thirst urgent—urine red and scanty. M. Fouquier was called into consultation, and after an attentive examination, pronounced the disease a violent fit of asthma, without any notable lesion of the thoracic organs. In this, with a trifling exception, M. Bricheteau agreed, and it was resolved to bleed from the arm—and reiterate sinapisms to different parts of the body. An anodyne emulsion was prescribed. The patient obstinately refused to be bled; but the other means were put in force. He was relieved. The breathing became more free—his mind more calm—and a sediment in his urine induced our author to hope that he would recover. But this calm was a false one. On the 20th, all the symptoms were intensely exasperated. His difficulty of breathing was insupportable—suffocation became imminent—and he could not rest a minute in one position. A blister to the thorax, and some trifling remedies. Next day the symptoms were still worse, and he insisted on being taken to a MAISON DE SANTÉ in a neighbouring street. He expired a few hours after he arrived there.

*Dissection.* The heart was rather larger than natural, and there were about two ounces of limpid serum in the pericardium. The right chambers of the heart were rather thin and somewhat enlarged, being filled with black blood and some fibrinous concretions. No morbid appearance in the valves or great vessels. The larynx and trachea presented no trace of disease; but the bronchia and their ramifications exhibited unequivocal marks of chronic inflammation, and were covered on their internal surface with a coat of thick mucus. The right lung was sound —but there were several portions of the left lung in a disorganized state; but still there was a great portion of it in a respirable condition, and the disease was of ancient date. There was no other lesion in any part of the body.

We have observed before, that certain continental writers, especially M. Rostan, consider asthma only a symptom of some organic lesion, generally of the heart or great vessels. M. Rostan appears to have been led to this conclusion by the examination of a number of old asthmatics, in whose bodies were almost always found traces of organic disease. But these changes were more likely to be the effect than the cause of the long-continued asthma. The very circumstance of asthmatic people being generally long-lived, militates most strongly against the organic theory—for where do we see long life under unequivocal disease of the heart or large vessels?

It appears to us, that a paroxysm of what is called and described as spasmodic or periodical asthma, is much akin to one of gout—that is, that there is an increase of irritation in the mucous membrane and, indeed, in the whole structure of the air-cells, with an afflux of blood to the parts—the consequence of which, is a constriction of the air-vessels and cells, with the laborious breathing so characteristic of the complaint. This state is relieved, after a certain period, by some discharge—generally an increased secretion from the mucous membrane of the lungs, and the paroxysm ceases, till some occasional cause or constitutional tendency renews the attack. In this way asthmatics go on for many years, and rarely die of the disease under which they have so long suffered. But there can be no doubt that these repeated orgasms in the system, tend ultimately to derange the function and even the structure of the heart and lungs—hence in old asthmatics, we may calculate on finding more or less of those organic lesions, which Rostan and others have too rashly pronounced the original cause of the asthmatic paroxysms.

